# Identification of nonclinical interventions for spontaneous recovery of depression using mathematical modeling

**DOI:** 10.1002/brb3.1550

**Published:** 2020-02-05

**Authors:** Gayathree Mohan, Dinesh Kumar Kandaswamy, Mohan Kumar Chikkaharohalli Ramakrishna

**Affiliations:** ^1^ Department of Physics MVJ Engineering College Bangalore India; ^2^ Department of Epidemiology and Public Health Central University of Tamilnadu Thiruvarur India; ^3^ School of Optometry and Vision Sciences Cardiff University Cardiff UK

**Keywords:** adolescent depression, clarity and reparation, emotional quotient variables attention, major depressive disorder, mathematical modeling, subjective well‐being, suicide ideation

## Abstract

**Background:**

In order to make a risk or vulnerability assessment of major depressive disorder (MDD) in adolescents and suggest nonclinical interventions for spontaneous recovery for low‐vulnerable adolescents a novel network mathematical model has been proposed.

**Methods:**

In the existing network theory, the theoretical model consists of a symptom network surrounded by the triggering factors as external field which are the cause for adolescents being diagnosed with MDD. But in our network model, the triggering external field is replaced by nonclinical interventions, easily implementable in schools and colleges with teachers as facilitators.

**Results:**

The four variables of subjective well‐being (SWB), emotional quotient—Attention (EQ‐A), emotional quotient—Clarity (EQ‐C) and emotional quotient—Reparation (EQ‐R) were the symptoms considered for stratification of the vulnerability. The mathematical model was created using the four symptoms and the four nonclinical interventions of technology use, physical exercise, peer pressure positive and peer pressure negative, and their inter‐relationship.

**Conclusion:**

A balance of tech use and physical exercise and of the peer pressure help maintain the adolescents in the low‐vulnerability group in our study with 227 adolescents in Bangalore. Furthermore, we predict that positive peer pressure and physical exercise could increase the EQ thus suggesting a preventive model for the onset of major depressive disorder (MDD).

## Graphical abstract

This article summarizes the causes of depression in a group of college students and also justifies the use of nonclinical intervention to reduce depression among adolescents. Mathematical model of symptoms and nonclinical interventions—A theoretical model based on subjective well‐being and three variables of Emotional Quotient namely Attention, Clarity, and Reparation. The 4 nonclinical interventions of ϵ, negative peer pressure, *δ*, positive peer pressure, *α*, rate of doing physical exercise; and *β*, rate of using technology govern the movement between the three vulnerabilities.

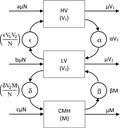

## 1 INTRODUCTION

One of the most common mental disorders that affect 121 million people worldwide is major depressive disorder (MDD). The World Health Organization has estimated that MDD will be the second major disability causing disease in the world by 2020 (Miller, Constance, & Brennan, 2007). The disability caused for adolescents is much more than adults as people with depression may experience lack of interest in daily activities, have poor concentration with low energy, have a feeling of worthlessness and so a lot of productive years are lost due to morbidity. Adolescent depression needs to be addressed early as it causes severe emotional damage leading to suicide, which is the second leading cause of death in this age group (Thapar, Collishaw, Pine, & Thapar, 2012). Twenty percent of the world's adolescents have a mental health or behavioral problems leading to MDD (Windfuhr et al., 2008). Obesity, substance abuse, increased rate of smoking, and social and educational impairments are serious risks if adolescent depression is not detected and treated early. In the Indian context due to rapid globalization and urbanization with breaking up of joint families and traditional support systems, the stress faced by these adolescents is enormous and their empowerment is extremely essential to increase their productivity. Early identification of MDD in adolescents can make a spontaneous recovery happen (Grover, Dutt, & Avasthi, 2010); otherwise, they undergo clinical interventions causing huge financial burden and productivity loss for both the family and country at large. Network theory has clear implications in understanding psychological diagnosis and treatment of MDD. The diagnosis involves identifying network of symptoms, and the treatment involves changing or manipulating the psychopathological network (McNally, 2016). One of the easiest ways to manipulate the psychopathological network is to create nonclinical interventions on symptoms thus deactivating them to create spontaneous remission. There are two different kinds of depression namely a nonclinical and a clinical depression. The nonclinical form is usually easier to treat because it is less severe and time bound and will end, with or without therapy with the depressed person usually benefitting from the therapy. On the other hand, clinical depression can be more severe, especially if it includes significant autonomic or vegetative symptoms. With these symptoms, the body really begins to shut down with no energy/no appetite/serious sleep disturbance and impaired thinking, concentration. These symptoms seldom respond to just psychotherapy so usually medication has to be added. Thus, clinical interventions are required when the MDD reaches or crosses a tipping point and are a huge drain in resources like time and money. But nonclinical interventions work better before MDD reaches the tipping point and so it becomes extremely important to assess a person's vulnerability to MDD. Novel mathematical modeling with MDD symptoms and nonclinical interventions supported by extensive real‐world data provide insights into the vulnerability of a given adolescent population thus dividing it into three states of high vulnerability, low vulnerability, and complete mental health (zero vulnerability). The main objective of creating the mathematical model was to recommend ways to remove or lessen the effect of MDD and to understand the impact of MDD over a given adolescent population. Early‐stage school‐based interventions can completely prevent the onset of MDD thus reducing the risk factors and increasing the protective factors to promote mental health and well‐being of adolescents.

What makes certain group of adolescents (Cramer et al., 2016) more vulnerable to develop MDD than others? From a network perspective, if the connection between the symptoms is activated and strong, the person is more vulnerable to develop MDD than a person whose symptoms are deactivated. We had to select the symptoms which caused MDD in a nonspecialist environment and instead of using the DSM‐IV manual to assess the presence of MDD with a psychologist, we relied on the study of well‐being in schools and colleges. One of the aims of study was to suggest nonclinical interventional measures in the current Promotive Mental Health and Well Being (PMHWB) programme, initiated from the District Administration, Kolar, Government of Karnataka (GoK) of promoting mental health of adolescents in schools (Vranda, 2015) using teachers as facilitators, to develop a manual for the teachers on the symptoms to look for and the nonclinical interventions that work best on the adolescents; test out the manual by training teachers as facilitators to implement the prevention and risk assessment of MDD program in the schools. As MDD completely affects the well‐being of an individual and his/her ability to handle and process emotions, we decided to include well‐being and emotional quotient (EQ) as the two most important parameters in our study. There are two kinds of well‐being namely subjective well‐being (SWB) and objective well‐being (Diener, Emmons, Larsen, & Griffin, 1985). Subjective well‐being is measured by the decision of only one person but objective well‐being is measured by more than one person's decision. There are three variables of EQ namely Attention to feelings (EQ‐A), Clarity of feelings (EQ‐C), and Mood repair (EQ‐R). Our stratification symptom network for vulnerability assessment was the four factors of SWB, EQ‐A, EQ‐C, and EQ‐R. Emotional intelligence (EI), which is measured in terms of Emotional Quotient (Zeidner & Olnick‐Shemesh, 2010), has been hypothesized to predict one's subjective sense of well‐being and positive mental health because high EI individuals are more aware of their emotions and better able to regulate them and so experience lower stress and higher levels of SWB. In the traditional network theory, there were three different ways of manipulating the psychopathological network, namely (1) interventions on the symptoms (modifying the status of one or more symptoms), (2) interventions in the external field eliminating the triggering causes, or (3) network interventions by modifying the connections between the nodes of the network. Instead of any one of the above 3 methods (Borsboom, 2017), we chose nonclinical interventions to replace the negative external field triggering MDD. The nonclinical interventions which replaced the external field in the traditional network theory were peer pressure (both positive and negative), technology use, and physical exercise. We selected physical exercise which is a positive factor as evidence shows that physical exercise (PE) is a strong epigenetic modulator (Mandolesi et al., 2018) that induces structural and functional changes in the brain, determining enormous benefit on both cognitive functioning and subjective well‐being. PE is “a sub classification of physical activity (PA) that is planned, structured, repetitive and has a final or an intermediate objective the improvement or maintenance of one or more components of physical fitness” according to WHO. A negative factor of technology use was selected in our clinical interventions as some researchers have associated online social networking with several psychiatric disorders, including depressive symptoms, anxiety, and low self‐esteem (Borsboom, 2017). Peer pressure has a strong influence on adolescents as it changes their mood, bringing about change in their knowledge, attitude, and practice, thereby leading to behavioral change communication (BCC). It can either be a positive influence or a negative influence depending on the nature of the influencing peer and their relation to the subjects. We created our mathematical model using four symptom variables and four of the clinical interventions and made a Jacobian analysis and found the reproduction number Ro (Riobello, 2015) to understand the rate of developing MDD in various adolescent groups. The basic reproduction number Ro is used to study the global impact that MDD can produce on an adolescent population.

## 2 MATERIAL AND METHODS

### 2.1 Ethical statement

This study was conducted at colleges in Bangalore between June 2018 and December 2018. The study protocol was approved by both the research and ethical committee of our institute in accordance with the principles stated in the Declaration of Helsinki. Written informed consent was obtained from each of the participants before participating in the study, and participants were anonymized by specific internal codes for analysis.

### 2.2 Participants and procedure

Participants in the study were 227 adolescents (99 females and 128 males) aged between 15 and 23 years (*M* = 18.58; *SD* = 0.29) all from Pre‐University colleges and engineering colleges in Bangalore. The assessment was carried out during class break time with informed consent and guarantee of anonymity for the participants with approval of the college authorities.

### 2.3 Instruments

Since we had to measure three different aspects of an individual like subjective well‐being, objective well‐being, and emotional quotient, we used 3 different questionnaires and combined them into a single entity to complete our study. The various components in our questionnaires were as follows:

1. Subjective well‐being is measured by questions such as “How satisfied are you with life as a whole?” This is measured by the satisfaction with life scale (SWLS) described in the literature (Diener et al., 1985).
2. The emotional quotient variable was measured using the Trait Meta‐Mood Scale (TMMS) (Salguero, Fernandez‐Berrocal, Balluerka, & Aritzeta, 2010). This was designed to assess how people reflect upon their moods and determine the extent to which people attend to and value their feelings (Attention). If they feel clear rather than confused about their feelings (clarity) and use positive thinking to repair negative moods (repair). It is answered using a 5‐point Likert scale, with options ranging from 1 = Strongly Disagree to 5 = Strongly Agree. The Trait Meta‐Mood Scale had different scoring levels for males and females and so we separated the study to check the differences between the two genders in the high‐vulnerability and low‐vulnerability population.
3. Material well‐being, health, longevity, literacy, and education are the constituents of the objective well‐being for which we chose socioeconomic status, social performance, and physical health questions in our questionnaire
4. Additionally, we wanted to study the effect of technology use and physical exercise in increasing or decreasing the risk of MDD.

**Figure 1:**
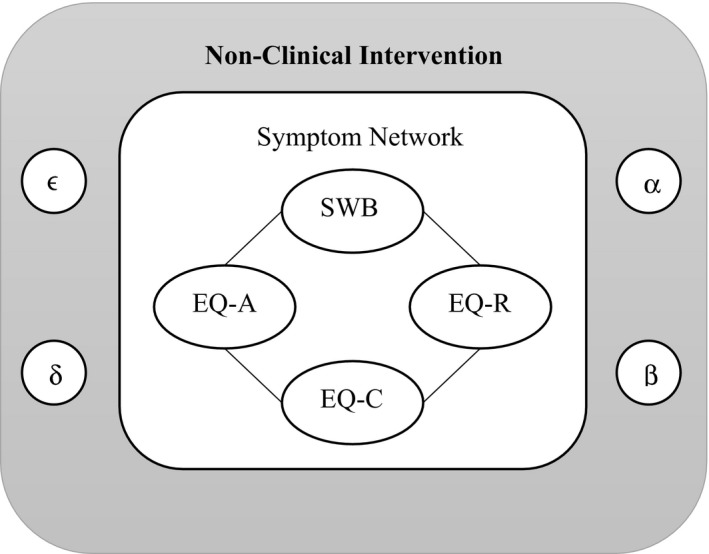
A Symptom network of 4 symptoms + 4 Nonclinical Interventions—Our novel theoretical network model where the symptom network with SWB and EQ variables is surrounded by nonclinical interventions instead of the force field causing the trigger in any psychological illness

**Figure 2:**
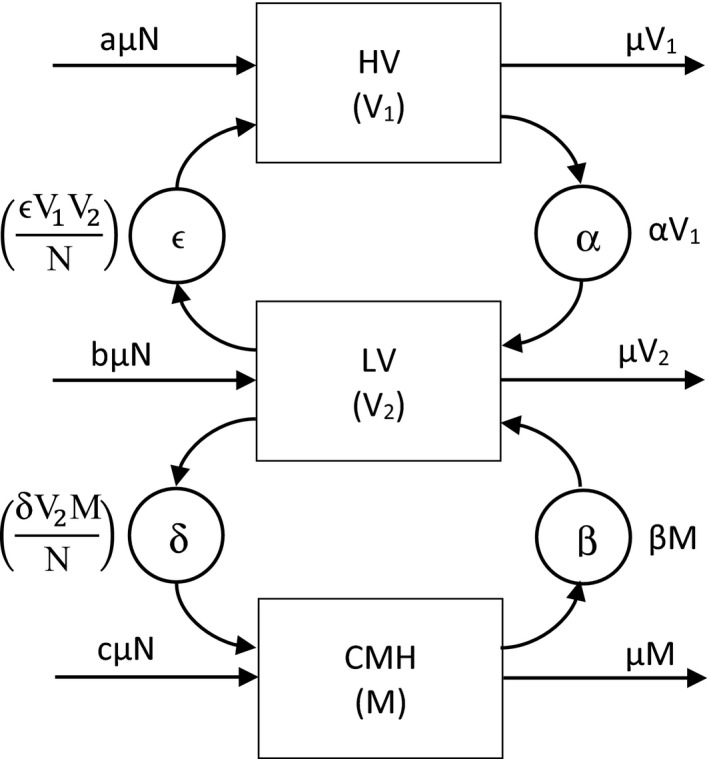
Mathematical model of symptoms and nonclinical interventions—A theoretical model based on subjective well‐being and three variables of Emotional Quotient namely Attention, Clarity, and Reparation. The 4 nonclinical interventions of *ϵ*, negative peer pressure; *δ*, positive peer pressure; *α*, rate of doing physical exercise, and *β*, rate of using technology govern the movement between the three vulnerabilities

A mathematical model has been evolved in order to segregate the population into three groups of high vulnerability, low vulnerability, and complete mental health (zero vulnerability) and study the movement between the groups. To divide our population into three groups of complete mental health (*M*), a low‐vulnerability population (*V*_1_), and a high‐vulnerability population (*V*_2_), we used the four factors of SWB, EQ‐A, EQ‐C, and EQ‐R. Each of these four factors has three different states of low, adequate/neutral, and high with separate scoring patterns for males and females in our population. Our novel network theory containing four symptomatic factors of SWB, EQ‐A, EQ‐C, and EQ‐R along with the four nonclinical interventions are shown in Figure 1. All the four symptoms are connected inside our network, and the four nonclinical interventions have replaced the external field factors like genetic, biological, and psychosocial factors, which trigger MDD. The difference here is that the external field factors push an individual into MDD but the nonclinical interventions that we have selected could make a spontaneous recovery without the aid of chemical antidepressants in the body. Two of our nonclinical interventions like the negative peer pressure and technology use are negative factors causing MDD and two others of physical exercise and positive peer pressure are positive factors, which reduce MDD. The scoring pattern of the four variables is shown in Table 1. So, a combination of superposed states of (3 states) * (4 variables) = 3^4^ = 81 states were made. All the 81 states had scores ranging from 80 to 140, and we stratified them into three groups based on their overall scores as shown in Table 2. There was only one state with a score above 120 falling into complete mental health group, 50 states in low‐vulnerability group, and 30 states in high‐vulnerability group. After using these 81 superposed states on 227 data points, 3 fell into the complete mental health group (*M*), 136 in the low‐vulnerability group (*V*_2_) and 88 in the high‐vulnerability group (*V*_1_). The movement between our three groups is studied using Figure 2.

**Table 1:** Scoring pattern of Stratification variables of SWB, EQ‐A, EQ‐C, and EQ‐R

SNo	SWB Score	SWB range name	EQ‐A score		EQ range name	EQ‐C score		EQ‐R score
			Male	Female		Male	Female	Male/Female
1	≥16	High	≥33	≥36	High	≥36	≥35	≥35
2	15	Neutral	22–32	25–35	Adequate	26–35	24–34	24–34
3	≤14	Low	≤21	≤36	Low	≤25	≤23	≤23

**Table 2:** Stratification 3 groups using 4 symptomatic variables of SWB, EQ‐A, EQ‐C, and EQ‐R

SNO	SWB	EQ‐A	EQ‐C	EQ‐R	Vulnerability	No. of States
1	Scores above 120				Complete mental health	1
2	Scores between 100 and 120				Low vulnerability	50
3	Scores below 100				High vulnerability	30

## 3 THEORY

### 3.1 Mathematical model assumptions

Let the total number of adolescents in our study (Salguero et al., 2010) in Bangalore be *N*. The per capita rate at which adolescents become 15 years to 23 years and enter our system is *µ*. This *µ* is also the rate at which they leave our study after completing 23 years. Adolescents in the low‐vulnerability group (*V*_2_) group move to high‐vulnerability group (*V*_1_) due to peer pressure from the high‐vulnerability group at the rate ϵ (negative peer pressure) pulling them upward to *V*_1_. Adolescents in low‐vulnerability (*V*_2_) group can move to the complete mental health group (*M*) due to peer pressure from the complete mental health at the rate of *δ* (positive peer pressure) pulling them downward to *M*. Technology use (negative factor) at the rate of *β* pushes the complete mental health (*M*) population into the low‐vulnerability group (*V*_2_). Physical exercise was a positive factor at the rate of α moving the high‐vulnerability population (*V*_1_) into the low‐vulnerability group (*V*_2_). Let *a*, *b*, and *c* be the probabilities of the total population moving into high risk, low risk, and complete mental health group, respectively. A list of the various parameters used in our model is shown in Table 3.

**Table 3:** List of Parameters used in the theoretical model

Parameter	Definition
*N*	Total adolescent population in Bangalore between ages 15 and 23
*µ*	Per capita rate at which adolescents enter and leave the system
*a*	Probability of being highly vulnerable to peer pressure at age 15
*b*	Probability of being less vulnerable to peer pressure at age 15
*c*	Probability of being with complete mental health at age 15
*δ*	Per capita peer pressure rate exerted by the complete mental class upon the low vulnerability class pulling them downward
*ϵ*	Per capita peer pressure rate exerted by the high vulnerability class upon the low vulnerability class pulling them upward
*α*	Per capita rate of doing physical exercise thereby reducing the risk from high vulnerability to low vulnerability.
*β*	Per capita rate of using technology thereby increasing the risk from the complete mental health group to the low‐vulnerability group.

### 3.2 Model equations

$$\frac{dV_{1}}{\mathrm{dt}}=a\mu N-\mu V_{1}+\frac{\epsilon V_{1}V_{2}}{N}-\alpha V_{1}$$

$$\frac{dV_{2}}{\mathrm{dt}}=b\mu N-\mu V_{2}-\frac{\delta V_{2}M}{N}-\frac{\epsilon V_{1}V_{2}}{N}+\alpha V_{1}+\beta M$$

$$\frac{\mathrm{dM}}{\mathrm{dt}}=c\mu N-\mu M+\frac{\delta V_{2}M}{N}-\beta M$$

Rescaling the above equations using $\frac{V_{1}}{N}=x\frac{V_{2}}{N}=y\;\text{and}\;\frac{M}{N}=z$ with x+y+z=1.

$$\frac{\mathrm{dx}}{\mathrm{dt}}=a\mu -\mu x+\epsilon xy-\alpha x$$

$$\frac{\mathrm{dy}}{\mathrm{dt}}=b\mu -\mu y-\epsilon xy+\alpha x-\delta yz+\beta z$$

$$\frac{\mathrm{dz}}{\mathrm{dt}}=c\mu -\mu z+\delta yz-\beta z$$

### 3.3 Equilibrium analysis

At equilibrium $\frac{\mathrm{dx}}{\mathrm{dt}}=\frac{\mathrm{dy}}{\mathrm{dt}}=\frac{\mathrm{dz}}{\mathrm{dt}}=0$.

cμ-μz+δyz-βz=0 from which $z=\frac{c\mu }{\beta +\mu -\delta y}$

Solving the first equation by using x=1-y-z and then substituting the value of *z* we have.

aμ+ϵxy-μx-αx=0

$$a\mu +\epsilon y\left(1-y-z\right)-\mu \left(1-y-z\right)-\alpha \left(1-y-z\right)=0$$

$$a\mu -\mu +\mu y+\epsilon y-\epsilon y^{2}-\alpha +\alpha y+\left(\mu +\alpha -\epsilon y\right)\frac{c\mu }{\mu +\beta -\delta y}=0$$

Which when simplified produces a cubic equation in *y* of the form.

$$Ay^{3}+By^{2}+Cy+D=0\;\text{where}$$

$$A=\epsilon \delta \;\;\;B=-\left(\mu \delta +\epsilon \delta +\epsilon \beta +\alpha \delta +\mu \epsilon \right)$$

$$C=\mu \delta -a\mu \delta +\mu^{2}+\mu \beta +\epsilon \mu +\epsilon \beta +\alpha \delta +\alpha \mu +\alpha \beta -c\epsilon \mu$$

$$D=a\mu^{2}+a\mu \beta -\mu^{2}-\mu \beta -\alpha \mu -\alpha \beta +c\mu^{2}+c\mu \alpha$$

We require the roots to be positive to give a realistic solution using Vieta's formula for a cubic equation:

$$\text{The sum of the roots}\;S_{1}=\frac{-B}{A}=\frac{\mu \delta +\epsilon \delta +\epsilon \beta +\alpha \delta +\mu \epsilon }{\epsilon \delta }$$

$$\text{Second sum of roots}\;S_{2}=\frac{C}{A}=\frac{\mu \delta -a\mu \delta +\mu^{2}+\mu \beta +\epsilon \mu +\epsilon \beta +\alpha \delta +\alpha \mu +\alpha \beta -c\epsilon \mu }{\epsilon \delta }$$

$$\text{Product of the roots}\;P=\frac{-D}{A}=\frac{-\left(a\mu^{2}+a\mu \beta -\mu^{2}-\mu \beta -\alpha \mu -\alpha \beta +c\mu^{2}+c\mu \alpha \right)}{\epsilon \delta }$$

For positive roots, we get the condition that *S*_1_, *S*_2,_ and *P* should be greater than zero.

$$\frac{\mu \delta +\epsilon \delta +\epsilon \beta +\alpha \delta +\mu \epsilon }{\epsilon \delta }>0\;\frac{\mu \delta -a\mu \delta +\mu^{2}+\mu \beta +\epsilon \mu +\epsilon \beta +\alpha \delta +\alpha \mu +\alpha \beta -c\epsilon \mu }{\epsilon \delta }>0$$

$$\frac{-\left(a\mu^{2}+a\mu \beta -\mu^{2}-\mu \beta -\alpha \mu -\alpha \beta +c\mu^{2}+c\mu \alpha \right)}{\epsilon \delta }>0$$

### 3.4 Jacobian analysis

We identify the equilibrium point as (0,0,1) which is the disease‐free equilibrium point and obtain the Jacobian at the equilibrium point to be.

$$J\left(0,0,1\right)=\left[\begin{array}{ccc} -\mu -\alpha & 0 & 0\\ \alpha & -\mu -\delta & \beta \\ 0 & \delta & -\mu -\beta \end{array}\right]$$

The eigenvalues of the Jacobian *J* (0,0,1) are found as follows.

$$\left|J\left(0,0,1\right)-\lambda I\right|=0$$

$$\left|\begin{array}{ccc} -\mu -\alpha -\lambda & 0 & 0\\ \alpha & -\mu -\delta -\lambda & \beta \\ 0 & \delta & -\mu -\beta -\lambda \end{array}\right|=0$$

$$\lambda_{1}=-\left(\mu +\alpha \right)\;\;\lambda_{2}=-\mu \;\;\lambda_{3}=-\left(\mu +\beta +\delta \right)$$

From the above three eigenvalues of the Jacobian matrix, we fix the endemic equilibrium point of our system as $R_{0}=-\left(\mu +\beta +\delta \right)$. The reproduction number $R_{0}$ is a manifestation of all the eigenvalues of the Jacobian matrix at the disease‐free equilibrium. In our case when *S*_1_ > 0, we have the following conditions on our various parameters.

μδ+ϵδ+ϵβ+αδ+μϵ>0

$$\delta \left(\mu +\alpha \right)+\epsilon \left(\mu +\beta +\delta \right)>0$$

$$\delta \left(\mu +\alpha \right)-\epsilon R_{0}>0$$

$$R_{0}<\frac{\delta }{\epsilon }\left(\mu +\alpha \right)$$

The value of the reproduction number depends on the ratio of our peer pressure terms. The more the value of the negative term ϵ the more the people in the high‐vulnerability group and the more the value of the positive term, δ the more the people in the complete mental health group.

All the eigenvalues of the Jacobian are negative so we have a stable node or focus as $R_{0}<1$. The equilibrium point (0,0,1) is asymptotically stable whenever *R*_0_ < 1.

### 3.5 Variable and parameter estimate from our data

After using these 81 superposed states on *N* = 227 data points, 3 fell into the complete mental health group (*M*), 136 in the low‐vulnerability group (*V*_2_) and 88 in the high‐vulnerability group (*V*_1_). The rescaled variables of $x=\frac{V_{1}}{N}=\frac{88}{227}=0.3876,\;y=\frac{V_{2}}{N}=\frac{136}{227}=0.5991,\;z=\frac{M}{N}=\frac{3}{227}=0.0132$.

1. The rate at which adolescents are introduced into the population per unit of time on an average is considered. It is measured as population per time or plain per time. The per capita rate *µ* of adolescents entering and leaving our study can be understood as follows. If (1/*µ*) is the average life span in the system, then our data with adolescents in ages 15–23 years with an average of 8 years or 8(365) days with $\mu =\frac{1}{8\left(365\right)}=\frac{1}{2920}=0.00342$. The unit of *µ* is thus/day.
2. We require the probability of adolescents being in the highly vulnerable group, a which can be calculated as follows. From our data, we see that $a\mu N=V_{1},\;a\mu =x=0.3876,\;a=\left(\frac{x}{\mu }\right)=\left(\frac{0.3876}{0.00342}\right)=\left(\frac{113.33}{365\;\text{days}}\right)=0.3104$. Similarly *b* = 0.4799 and *c* = 0.0105.
3. *δ* is the product of two terms one being the number of contacts that a person in the low‐vulnerable group *V*_2_ has with individuals in the complete mental health group M and the other being the “correction probability” of these contacts. The correction probability is a conversation of at least 5 min about positive outcomes. We assume that there are about 5 such contacts per day and that 10 of them are required for “correction” of a person into a positive frame of mind thus decreasing his chances of getting into MDD. Hence, *δ* = 5(1/10) = (1/2) = 0.5.
4. ϵ is also the product of two terms one being the number of contacts that a person in the highly vulnerable group *V*_1_ has with individuals in the low‐vulnerable group *V*_2_ and the other being the “infection probability” of these contacts. The infection probability is a conversation of at least 5 min about negative outcomes capable of pushing the adolescent into MDD. We estimate that a highly vulnerable individual has about 5 such contacts per day and that 25 contacts are necessary for “infection” meaning MDD. Accordingly, ϵ = 5(1/25) = (1/5) = 0.2.
5. *α*, the rate of doing physical exercise, causes the population to move from high‐vulnerability group to the low‐vulnerability group. One hour of physical exercise per day was considered as the high‐exercise group with half an hour as the medium‐exercise group and 0.15 hr as the low‐exercise group. We found that in the high‐vulnerability group, people doing high exercise were 32, medium exercise were 13 and low exercise were 43 thereby calculating a weighted mean as $hr=\frac{32\times 1+13\times 0.5+43\times 0.15}{32+13+43}=0.5108\;\text{hr}$. For the low‐vulnerability group, people doing high exercise were 60, medium exercise were 20 and low exercise were 56 thereby with a weighted mean as $lr=\frac{60\times 1+20\times 0.5+56\times 0.15}{60+20+56}=0.5765\;\text{hr}$. The average of *hr* and *lr* value = (0.5108 + 0.5765)/2 = 0.54365 hr per day. Assuming he does the same level of physical exercise for 30 days continuously, then he moves from the high‐vulnerability group to the low‐vulnerability group. So *α* = (0.54365 × 30 days)/(24 hr) = 0.6796 per day.
6. Similarly, *β*, the rate of using technology, causes the population to move from complete mental health to low‐vulnerability group. Two hours of technology use per day was considered as the high‐tech group with one hour as the medium‐tech group and 0.30 hr as the low technology use group. We found that in the complete mental health group, people with high technology use were 1, with medium technology use were 0 and with low technology use were 2 thereby with a weighted mean as $cmh=\frac{2\times 1+1\times 0+0.3\times 2}{1+0+2}=0.8666\;\text{hr}$. For the low‐vulnerability group, people with high technology use were 63, medium technology use were 52 and low technology use were 21 with a weighted mean as $lrt=\frac{63\times 2+52\times 1+21\times 0.3}{63+52+21}=1.3551\;\text{hr}$. The average of *cmh* and *lrt* value = (0.8666 + 1.3551)/2 = 1.1110 hr per day. Assuming that he does the same level of technology use for 15 days continuously then he moves from the complete mental health group to the low‐vulnerability group. So *β* = (1.1110 × 15 days)/(24 hr) = 0.6941 per day. The values of the variables and parameters used in the mathematical model are presented in Tables 4 and 5.

**Table 4:** Variable initial values in the theoretical model

Variable names	*x*	*y*	*z*
Variable initial values	0.3876	0.5991	0.0132

**Table 5:** Parameter estimates in the theoretical model

Parameter name	*µ*	*a*	*b*	*c*	*δ*	*ϵ*	*α*	*β*
Parameter estimate	0.00342	0.3104	0.4799	0.0105	0.5	0.2	0.6796	0.6941

## 4 RESULTS

**Figure 3:**
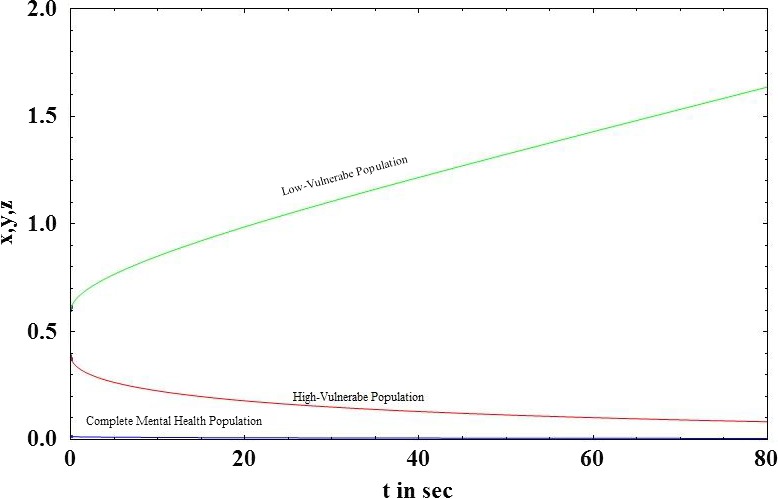
Stabilization of the highly vulnerable, low‐vulnerable and complete mental health population with respect to time—The variation of the rescaled variables of *x* = (*V*_1_/*N*); *y* = (*V*_2_/*N*); *z* = (*M/N*) with respect to time in seconds is seen in figure. The figure shows the increase in the low‐vulnerable population and decrease in the highly vulnerable population and complete mental health reaching a steady state after some time *β*

**Figure 4:**
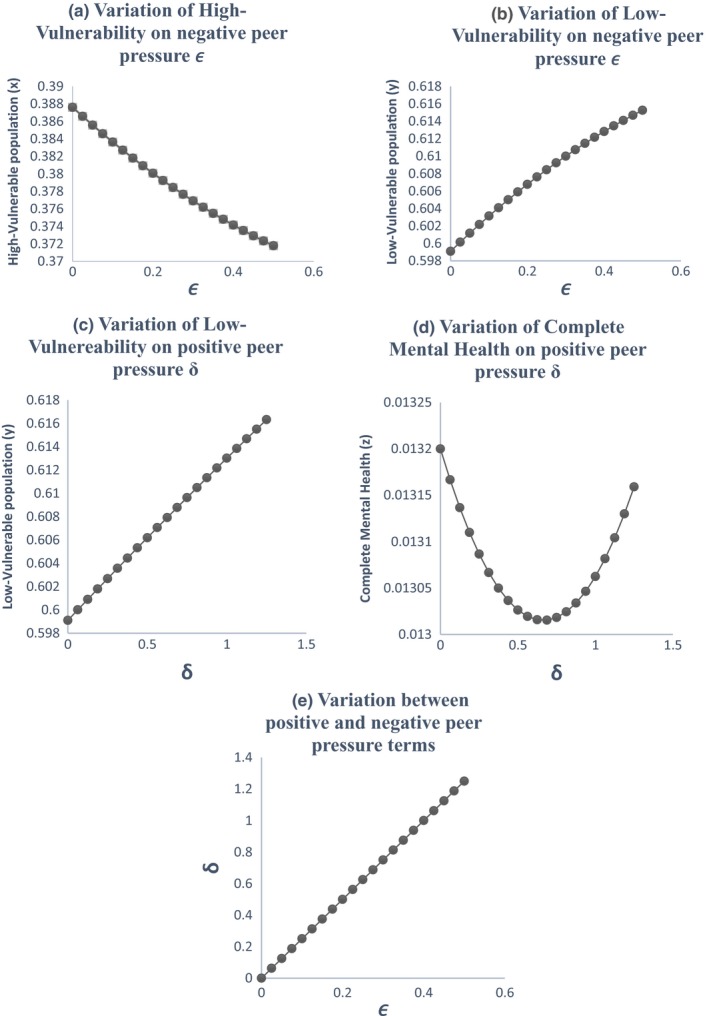
Effect of positive and negative peer pressure on different groups—Since the negative peer pressure *ϵ* is between high vulnerability and low vulnerability, we study its variation with *ϵ* to find that as the negative peer pressure increases the highly vulnerable population decreases as in (a) and the low‐vulnerable population increases as in (b). Since the positive peer pressure *δ* is between low vulnerability and complete mental health, we study its variation with *δ* to find that as it increases the low vulnerable population increases and the complete mental health shows a decrease and then a further increase in (c) and (d). The variation of the two peer pressure terms both positive and negative shows a linear relationship in (e)

**Figure 5:**
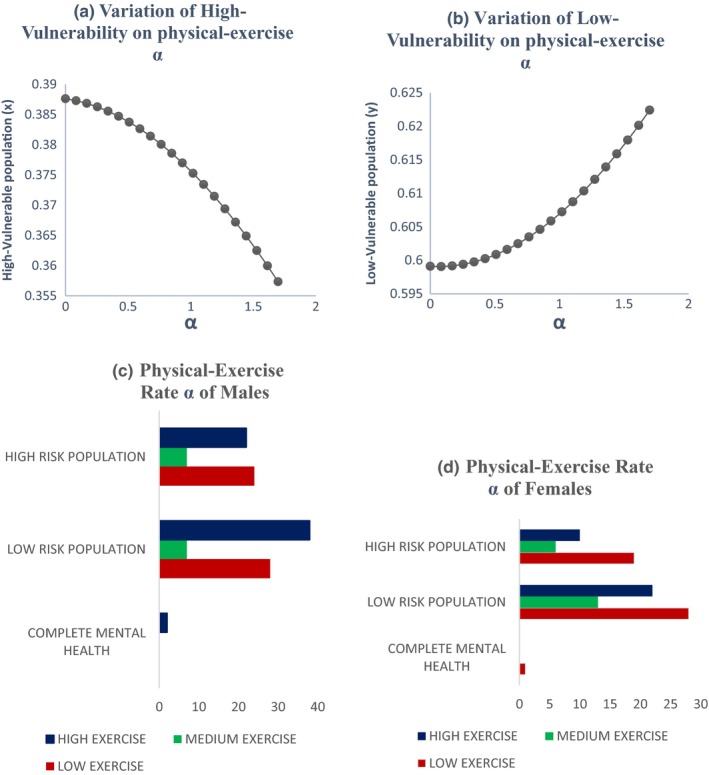
Impact of physical exercise between the high‐ and low‐vulnerable groups for depression with gender disparity—The rate of doing physical exercise α connects the highly vulnerable and low‐vulnerable population, so its variation between high vulnerability and rate of physical exercise shows that as α increases the highly vulnerable population decreases as seen in (a) and as *α* increases the low vulnerable population increases as shown in (b). (c) and (d) show the rate of doing physical exercise of both the male and female population in our cohort of 227 adolescents in Bangalore

**Figure 6:**
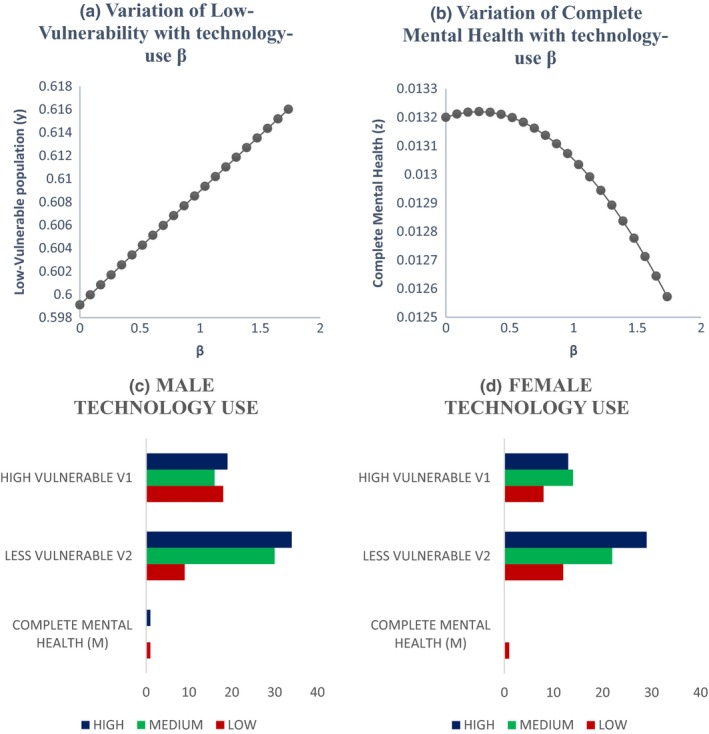
Impact of technology usage between the high‐ and low‐vulnerable groups for depression with gender differences—The technology use *β* connects low vulnerable population and the complete mental health group and so its variation between low vulnerability and *β* shows a linearly increasing line as in (a) and as *β* increases complete mental health group increases and then decreases as shown in (b). (c) and (d) show the rate of technology use of both the male and female population in our 227‐adolescent cohort

**Figure 7:**
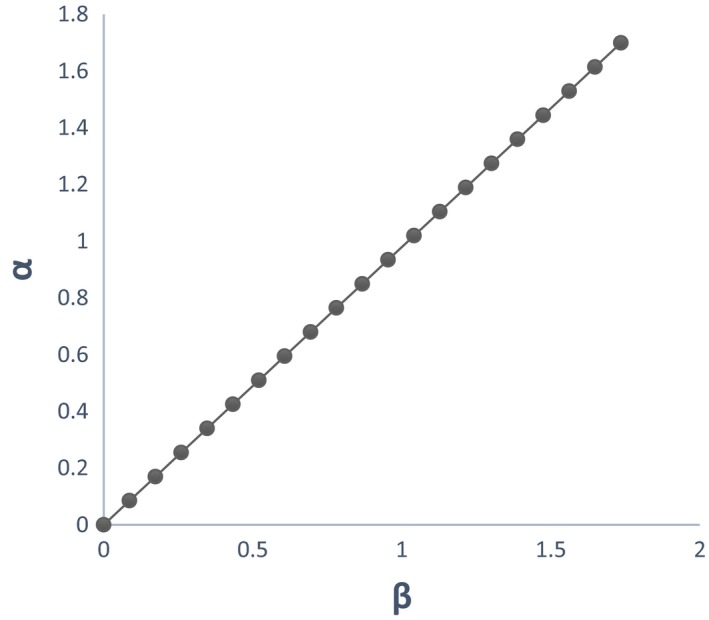
Variation between physical exercise rate and technology use rate—The variation between the rate of doing physical exercise α and the rate of technology use *β* is a straight line

**Figure 8:**
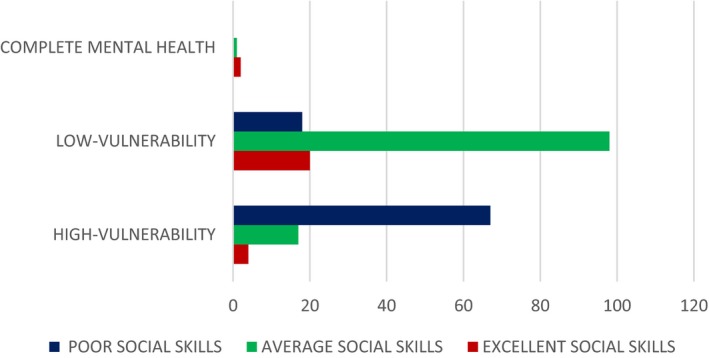
Social skills of our 3 group population—The social skills of our entire cohort of 227 adolescents in all the 3 vulnerable groups that we have classified has been shown in this figure

1. Graphs between *x*, *y*, *z*, and *t* are shown in Figure 3. All three vulnerable populations stabilize after a certain period of time. As per our theoretical model, more population fall into the low‐vulnerable group due to interplay of positive and negative peer pressure along with technology use and physical exercise. So, we see that the low‐vulnerable group stabilizes after increasing while the high‐vulnerable group and complete mental health group stabilize after decreasing. The initial values of the variables are taken from Table 4.
2. Effect of the peer pressure terms of *ϵ* and *δ*: The peer pressure term *ϵ* is a negative effect on the low‐vulnerability group population as it pushes them into the high‐vulnerability group after a few interactions or conversations contributing to negative outcomes. On the other hand, the peer pressure term *δ* is the positive influence with conversations on positive outcomes played by the population with complete mental health on the low‐vulnerability group there by bringing them to the complete mental health group. Variation of the high‐vulnerability population with ϵ is shown in Figure 4a and that of low‐vulnerability population is shown in Figure 4b. As the negative peer pressure term ϵ increases, the high‐vulnerable population decreases in Figure 4a which is contrary to our assumption that the more the interaction with negative people the more is the vulnerability to develop MDD making the population in high vulnerability more. Here, an opposite trend is shown as the other factors interplay into the negative peer influence. The same is the case with Figure 4b where because of negative peer influences the people in low vulnerability should decrease. We graph the two peer pressure influences one positive and the other negative on the low‐vulnerability population, complete mental health in Figure 4c, d, and obtain a linear relationship between them in Figure 4e. Figure 4d shows that the influence of peer pressure on complete mental health decreases the population initially and after the peer pressure term *δ* reaches a threshold value of 0.6875, the complete mental health population starts to increase. Figure 4(e) shows a linear relation between the two peer pressure terms *δ* and *ϵ* with the values of *δ* being almost double that of ϵ showing that the good influence should have a much higher value than the bad influence for a person to fall into complete mental health group.
3. Effect of physical exercise α: Increase in the rate of doing physical exercise α pushes the high‐vulnerability group into the low‐vulnerability group. A graph between *x*, *y*, and *α* shows the effect of this parameter in Figure 5a, b. As the rate of doing physical exercise increases (*α* increases), the high‐vulnerability population decreases, a person comes out of depression or its risk as shown in Figure 5a. Figure 5b on the other hand shows that as the rate of doing physical exercise increases, the number of people in the low‐vulnerability group increases. The highest exercise is observed in the male low‐vulnerability population (Figure 5c), and the lowest exercise is seen in the low‐vulnerability female population (Figure 5d). This shows that adolescent females do less physical exercise than adolescent males whether they are in high vulnerability or low vulnerability in our population study. Even in the complete mental health group, the females fall into low‐exercise group but the males fall into the high‐exercise group.
4. Effect of technology use *β*: The effect of technology use was individually analyzed for males and females, and we observed that the technology use is high in both highly vulnerable group as well as low‐vulnerable group compared with the complete mental health group. This shows that technology use has a negative impact on the mental health, probably because of the addiction it creates due to repeated usage and craving to use more (Figure 6). From the graph (Figure 6c), we see that the percentage of males with high tech use is 35% in high‐vulnerable group and 46% in the low‐vulnerable group. This result defies the common perception that high‐tech users would be more vulnerable to depression. But when we consider the other factors into play like physical exercise and peer pressure the result changes. Facilitating relationship maintenance and making new social connections are some of the functions of social networking sites (SNS). Either SNS can influence a person in the right direction (freedom of expression in thoughts feelings and identity, making new friendship) or it might end up in a wrong direction as well (miscommunication, maladaptive tendency, and social isolation) (Dillon, Baeza, Ruales, & Song, 2002). Relationship between complete mental health and technology use shows that as the technology use factor of *β* increases the complete mental health population decreases and the population moves to the low‐risk group (Figure 6b). Based on our results, the technology use of both males (Figure 6c) and females (Figure 6d) is high in the low risk more than the other two groups in our three‐group population. The high‐vulnerable female population has a medium technology use but the high‐vulnerable male population has a high technology use than the females.
5. Relation between the technology use and physical exercise shows a linear relationship (Figure 7a). Hence, high technology use might be compensated by the effect of high exercise as well, low exercise might compensate the effect of low technology use and thus nullify its positive effect.
6. Relation between social skills for our three‐group population is shown in Figure 8. As expected, the high‐vulnerable population has poor social skills and the complete mental health group has excellent social skills. Between males and females in our study, the males possessed average social skills as compared to the females who possessed excellent social skills.

## 5 DISCUSSION

Network analysis computes metrics of node centrality (Borsboom, 2017) instead of focussing on the symptoms unique to a certain mental disorder. Highly central nodes of degree, strength, expected influence, and closeness are of importance in the network.

1. *Degree centrality* is defined as the number of edges connected to a node and the higher the degree, the more central the node is to the network. For example, a node with many edges is like a person with many friendship connections. So, the more edges or friendships he has the lesser is his risk of developing MDD. In our survey, we see that the highly vulnerable population has poor social skills than the less‐vulnerable individuals who have an average social skill as shown in Figure 8. As expected, the complete mental health group has the maximum in excellent social skills. Our results are substantiating WHO motto of reducing depression through peer counseling, which is clearly mentioned by WHO in their Health slogan (2017) “Depression, Let's talk”. Half of the stress will go off, when it is discussed with a peer with positive coping skills. Also, between males and females, the females had much better social skills than the males in our study which might be attributed to the high use of technology by the males. Generally, the increased use of social networking sites (SNSs) would alienate males from real‐world social skills.
2. Activation of various symptoms in our network is reflected by maximizing *strength centrality* in the psychopathology network leading to an increased MDD. Swift recovery from an episode of MDD is possible if clinical interventions target on a symptom with high strength centrality. Between our selected symptoms of SWB and EQ, we find that EQ has high strength centrality and so strategies to increase the EQ of an individual would hasten the recovery process of the individual. Inculcating a desire to learn and grow and adopting a positive attitude in life would increase the Attention variable of EQ. Simple techniques, like meditation, empathy, and listening to other point of view draining your emotions toward inanimate objects, would improve the clarity variable of EQ. Counting backwards to calm down your mind when in anger or disturbed mood helps in mood repair variable of EQ, thereby stabilizing individuals toward complete mental health or zero vulnerability.
3. *Expected influence* takes into account both negative and positive edges. Simulations indicated that this new centrality metric matches the performance of strength centrality when networks contain only positive edges, but outperforms it as networks contain increasingly more negative edges. In our study, we use the peer pressure terms both positive and negative on the high‐vulnerable and low‐vulnerable population.
4. *Closeness centrality*: The closeness of a node is the average distance from that node to the other nodes in the network. Closeness is the inverse of farness. This metric is less useful in psychopathology than in epidemiology where infection of a person (node) high on closeness centrality will be more likely to incite a rapidly developing epidemic than will infection of a person low on closeness centrality. In our study, the duration of the contacts and the number of times the contacts happened is defined for the spread of infection.
5. Physical exercise (PE) is known to cause structural and functional alterations in the brain leading to substantial biological and psychological benefits (Zeidner & Olnick‐Shemesh, 2010). Some of the benefits of physical exercise include effective cognitive functions such as improved memory, efficient attention, and better control over processes (Chieffi et al., 2017; Seabrook, Kern, & Rickard, 2016). Moreover, physical exercise has shown to increase the academic performance in children and adults doing it on a regular basis compared with sedentary individuals of the same age (Donnelly et al., 2016; Voss, Nagamatsu, Liu‐Ambrose, & Kramer, 2011). In addition to all these factual findings, a recent study employing PET (positron emission tomography) imaging has demonstrated that PE establishes alteration in the metabolic network pertaining to cognition (Huang, Fang, Li, & Chen, 2016). Hence, it made us test the influence of this factor in the three different groups and our results show that physical exercise had a positive effect in bringing down depression.
6. The role of physical exercise and its impact on depression have been analyzed and we found that as physical exercise rate (*α*) increases, there is a decrease in the high‐vulnerability group and increase in the low‐vulnerability group (Figure 5). The current study shows that physical exercise has a crucial role in bringing an individual out from high vulnerability to low vulnerability as observed in other studies in the literature (Craft & Perna, 2004; Donnelly et al., 2016; Huang et al., 2016; Voss et al., 2011). Comparison of males and females pertaining to physical exercise shows that females tend to do less physical exercise than males, in spite of being in high‐vulnerable or low‐vulnerable or complete mental health group, whereas males show high physical exercise in all the three groups, hence, mental health of women can be enhanced by doing physical exercise. Lack of physical exercise by adolescent females can be cited as one of the reasons for them to be more prone to the risk of depression than the males in the category. This can be corrected by encouraging females to do more physical exercise by advertising special ladies’ time in existing gym facilities and compulsorily sending females to play during the PT (physical training) period in schools and colleges instead of converting all such PT periods into academic hours. Such a policy change by schools and colleges can drastically reduce adolescent females entering the high‐vulnerability group for major depressive disorder.
7. We were keener to know, how the individuals in high‐exercise group get into high‐vulnerable group of depression, we found that, though certain individuals do rigorous exercise, their excess/high technology use makes them prone to be in high‐vulnerability group. Similarly, we found, that even though certain individuals spend more time on technology usage, their higher rate of doing physical exercise makes them fall under the category of complete mental health. We could also observe that certain individuals are not affected by high technology use, though they do limited exercise that could be attributed due to the positive effect of social networking as discussed earlier.

## 6 CONCLUSION

In conclusion, our novel mathematical modeling using network theory has enabled us to identify the key symptoms and nonclinical interventions that could be implemented using teachers and counsellors as facilitators using simple questionnaires. The network mathematical model also helps in making risk assessment for prevention of MDD or for early interventions to influence the adolescents into the right direction to stay in complete mental health group with zero vulnerability for MDD. A major limitation in our study was that the internal factors such as predisposing genetic factors, childhood trauma, and triggering MDD were not considered in our mathematical model and so the diagnosis of endogenous depression, which arises as a result of an internal stressor cognitive or biological and not an external factor, is not ruled out. Endogenous depression often arises more in a positive family history of disorders and lesser due to psychosocial and environmental factors causing their symptoms. Future studies could include both the internal and external field factors along with nonclinical interventions in the mathematical model, so that we can predict if the nonclinical interventions can completely nullify or eradicate the adverse internal/external field factors.

## CONFLICTS OF INTERESTS

There are no conflicts of interests to be disclosed by any of the abovementioned authors.

## Data Availability

The data that support the findings of this study are available from the corresponding author upon reasonable request.
